# A Novel, Nonaquatic Zoonotic Transmission of *Mycobacterium marinum*

**DOI:** 10.1155/crdi/2767290

**Published:** 2024-12-27

**Authors:** Georgios Kravvas, Nada Aboukhatwah, Lola Meghoma, Victoria Vilenchik, Jon Oxley, Daniel Keith

**Affiliations:** ^1^Department of Medicine, University College London Hospitals, London, UK; ^2^Department of Dermatology, University College London Hospitals, London, UK; ^3^Department of Dermatology, Whittington Health NHS Foundation Trust, London, UK; ^4^Department of Dermatology, North Bristol NHS Trust, Bristol, UK; ^5^Department of Histopathology, North Bristol NHS Trust, Bristol, UK

**Keywords:** case report, granuloma, infectious diseases, *Mycobacterium* infections, *Mycobacterium marinum*, zoonotic

## Abstract

**Introduction:** *Mycobacterium marinum* was first described in humans in 1954, known to infect fish species and contaminate water and fish products. Inoculation to humans occurs through injured skin resulting in the formation of a solitary nodule known as “fish tank granuloma.” Disseminated infections have been reported in the immunocompromised and can present with extracutaneous manifestations such as arthritis and osteomyelitis. Nonaquatic transmission has not been previously reported.

**Case Presentation:** A 63-year-old lady with rheumatoid arthritis and bronchiectasis was referred to dermatology with widespread soft dermal plaques, pustules, erosions, and necrotic wounds. Multiple bacterial and viral swabs were negative. A biopsy was performed that showed a neutrophilic dermatosis suggestive of Sweet's Syndrome. The patient initially improved with prednisolone, but subsequently deteriorated with a worsening rash, joint pains, and neutropenic sepsis. Repeat biopsies eventually revealed abundant acid-fast bacilli, later confirmed to be *Mycobacterium marinum*. Our patient had no history of exposure to aquatic organisms but had previously cared for an inland bearded dragon with an unknown illness. Although infection with *M. marinum* has been reported in reptiles, cases of nonaquatic zoonotic transmission have not been described in the literature.

**Conclusion:** *Mycobacterium marinum* infection should be considered in patients with indicative clinical and histological features, especially in the immunocompromised, even in the absence of an obvious aquatic source of infection. Awareness of this entity could lead to earlier diagnoses and reduced morbidity and mortality.

## 1. Introduction

Linell and Norden first described *Mycobacterium marinum* in humans in 1954. The mycobacteria were isolated from the skin lesions of swimmers who had bathed in a contaminated pool [1]. *M. marinum* can infect many fish species, leading to contamination of water and fish products [2]. Inoculation to humans occurs through injured skin, resulting in the formation of a solitary nodule known as the “fish tank granuloma” [2]. The bacteria can spread via the lymphatics, in a sporotrichoid-like fashion, leading to further verrucous plaques and nodules, typically involving the hands [2]. Disseminated infection has been reported in the immunocompromised and can present with extracutaneous manifestations such as arthritis and osteomyelitis [3, 4].

### 1.1. Case Presentation

A 63-year-old Caucasian woman presented with a 6-week history of low-grade fever and widespread pustules that were resistant to treatment with flucloxacillin. Bacterial and viral swabs were performed and found to be negative. Blood tests showed mildly raised inflammatory markers but were otherwise unremarkable. The patient had a history of rheumatoid arthritis and bronchiectasis. Her medications included long-term methotrexate and intermittent courses of prednisolone.

On examination, she was found to have multiple scattered plaques, pustules, erosions, and necrotic wounds in a widespread distribution.

A skin biopsy was performed and was found to exhibit numerous pustules and intraepidermal neutrophils. The entire dermis and superficial subcutis showed heavy neutrophilic infiltration and necrotic debris within an apparent abscess. A diagnosis of a pustular variant of Sweet's syndrome was considered and treatment with prednisolone was commenced with some initial improvement of the symptoms.

**Figure 1:**
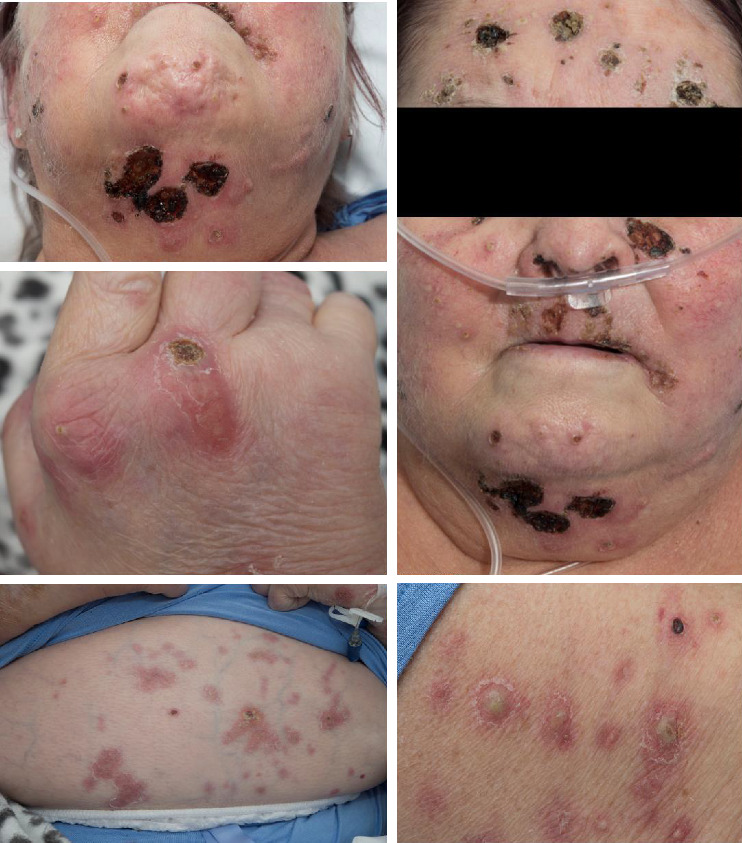
Widespread eruption with necrotic areas, pustules, and erythematous plaques.

Four months later the patient was admitted to hospital due to a worsening eruption with increasing pustules, necrotic areas, and erythematous plaques (Figure 1), as well as joint pains and neutropenic sepsis.

A repeat skin biopsy was performed that confirmed the previous findings and did not show any new features. The patient's condition did not improve following an increased dose of prednisolone, and treatment with Dapsone was therefore also commenced.

**Figure 2:**
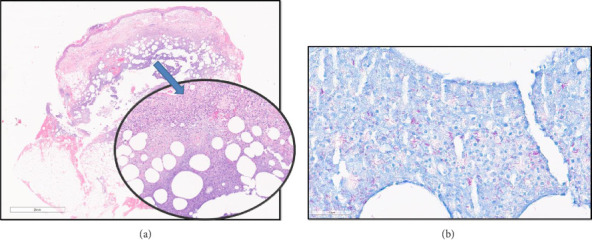
A. Haematoxylin and eosin and B. ZN-stained histopathology samples demonstrating: (a) Necrosis in the subcutaneous fat which is surrounded by neutrophils. (b) Numerous acid-fast bacilli in the areas of necrosis.

Despite that, the patient continued to deteriorate, and a third skin biopsy was performed. This time, histological examination demonstrated a necrotic abscess with deep dermal neutrophilic infiltration (Figure 2). The amount of infiltration and degree of fatty involvement was beyond that expected for Sweet's syndrome. Ziehl-Neelsen (ZN) staining was performed which revealed abundant atypical acid-fast bacilli (AFB).

ZN staining of earlier biopsies was performed retrospectively that also confirmed the presence of AFB. A fourth biopsy was undertaken for tissue culture, which isolated *Mycobacterium marinum*. Based on this information, the diagnosis was revised to disseminated *M. marinum* infection.

Following close questioning, the patient denied any contact with obvious aquatic sources of infection. She did, however, admit to having cared for a pet lizard (inland bearded dragon, *Pogona vitticeps*) that had died from an unknown illness shortly before the patient's diagnosis.

**Figure 3:**
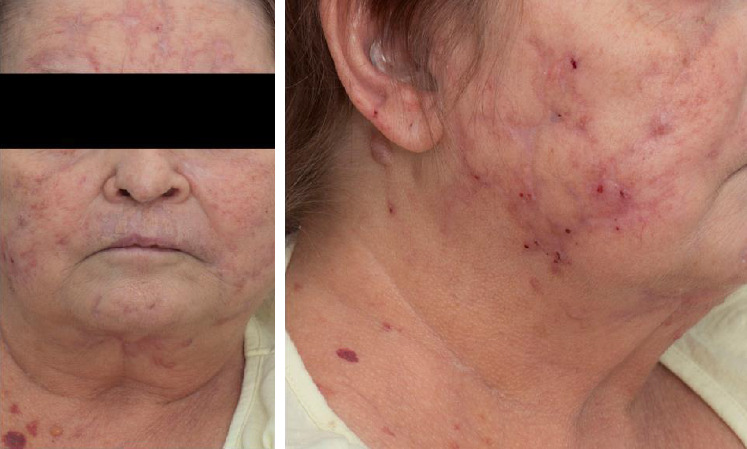
Photographs of the patient showing resolution of all cutaneous lesions with residual scarring and disfigurement.

The patient received multiple courses of antimycobacterial regimens with eventual resolution, albeit with significant scarring and disfigurement (Figure 3).

## 2. Discussion

Infection with *M. marinum* follows contact with infected water or via contact with an infected fish tank or shellfish [4]. The two conditions thought to be required for *M. marinum* infection are thought to be injury or abrasion to the skin and exposure to a contaminated aqueous environment. In one study, almost half (49%) of *M. marinum* infections were aquarium-related, 27% were related to fish or shellfish injuries, and 9% were related to injuries associated with saltwater or brackish water [5].

Disseminated cases are fairly uncommon. A small number of cases of *M. marinum* infection occurring in patients with HIV and in patients treated with tumor necrosis factor alpha (TNF-*α*) inhibitor have been reported [6]. Our patient's history of iatrogenic immunocompromise (regular methotrexate for rheumatoid arthritis, and intermittent prednisolone for bronchiectasis) is the likely culprit for her disseminated disease.

The inland bearded dragon is the most popular pet lizard in the United Kingdom and United States [7]. Systemic infection with *M. marinum* in an inland bearded dragon has been reported following the lizard's ingestion of infected guppies [7]. Other reptiles reported to be infected with *M. marinum* include the royal python *(Python regius)*, crocodilians, and Egyptian spiny-tailed lizards [7]. However, nonaquatic, zoonotic transmission of *M. marinum* has not been reported to date.

Interestingly, we could not elicit any history of contact between our patient and any aquatic sources of infection. The patient's exposure to an inland bearded dragon that died from an unknown illness near the time of diagnosis raises fascinating questions about the possibility of transmission via a surmised nonaquatic route. Although microbiological studies of the deceased dragon would have helped in testing this hypothesis, the body of the deceased dragon was unfortunately not available for testing.

The histology of *M. marinum* infected tissues often reveals the development of noncaseating granulomata from a neutrophilic abscess [8]. Few bacilli can be observed except in the immunocompromised host [8]. The surrounding dermis shows a lymphohistiocytic infiltrate, whilst the overlying epidermis can be ulcerated, parakeratotic, or acanthotic [8]. Overall, features are rather nonspecific with pseudoepitheliomatous hyperplasia accompanied by a poor yield of positive isolates from culture specimens [8].

We argue that a differential diagnosis of *M. marinum* infection should be considered in patients with indicative clinical and histological features, especially in the background of immunocompromise, despite the absence of an obvious aquatic source of infection. Awareness of this entity could lead to earlier diagnoses and reduced morbidity and mortality.

## Data Availability

No underlying data were collected or produced in this study.

## References

[1] Linell F., Norden A. (1954). Mycobacterium Balnei, a New Acid Fast Bacillus Occurring in Swimming Pools and Capable of Producing Skin Lesions in Humans. *Acta Tuberculosea Scandinavica Supplementum*.

[2] Schliemann S., Rässler F., Tittelbach J., Kranzer K., Zollmann C., Elsner P. (2018). Disseminated Mycobacterium Marinum Skin Infection Due to Chronic Lymphedema in an Immunocompetent Patient. *Journal of the German Society of Dermatology*.

[3] Gombert M. E., Goldstein E. J., Corrado M. L. (1981). Disseminated Mycobacterium Marinum Infection After Renal Transplantation. *Annals of Internal Medicine*.

[4] Gluckman S. (1995). Mycobacterium Marinum. *Clinical Dermatology*.

[5] Akram S. M., Aboobacker S. (2023). *Mycobacterium Marinum Infection*.

[6] Travis W. D., Travis L. B., Roberts G. D. (1985). The Histopathologic Spectrum in Mycobacterium Marinum Infection. *Archives of Pathology and Laboratory Medicine*.

[7] Girling S. J., Fraser M. A. (2007). Systemic Mycobacteriosis in an Inland Bearded Dragon (*Pogona vitticeps*). *Veternary Record*.

[8] McKee P. H., Calonje E., Grantner S. R. (2005). *Pathology of the Skin: With Clinical Correlations*.

